# Persistent Symmetry Frustration in Pollen Tubes

**DOI:** 10.1371/journal.pone.0048087

**Published:** 2012-11-05

**Authors:** Mariusz Pietruszka, Marcin Lipowczan, Anja Geitmann

**Affiliations:** 1 Laboratory of Plant Physiology, Faculty of Biology and Environmental Protection, University of Silesia, Katowice, Poland; 2 Department of Biophysics and Morphogenesis of Plants, Faculty of Biology and Environmental Protection, University of Silesia, Katowice, Poland; 3 Institut de Recherche en Biologie Végétale, Département de sciences biologiques, Université de Montréal, Montreal, Québec, Canada; University of Zurich, Switzerland

## Abstract

Pollen tubes are extremely rapidly growing plant cells whose morphogenesis is determined by spatial gradients in the biochemical composition of the cell wall. We investigate the hypothesis (MP) that the distribution of the local mechanical properties of the wall, corresponding to the change of the radial symmetry along the axial direction, may lead to growth oscillations in pollen tubes. We claim that the experimentally observed oscillations originate from the symmetry change at the transition zone, where both intervening symmetries (cylindrical and spherical) meet. The characteristic oscillations between resonating symmetries at a given (constant) turgor pressure and a gradient of wall material constants may be identified with the observed growth-cycles in pollen tubes.

## Introduction

**Figure 1:**
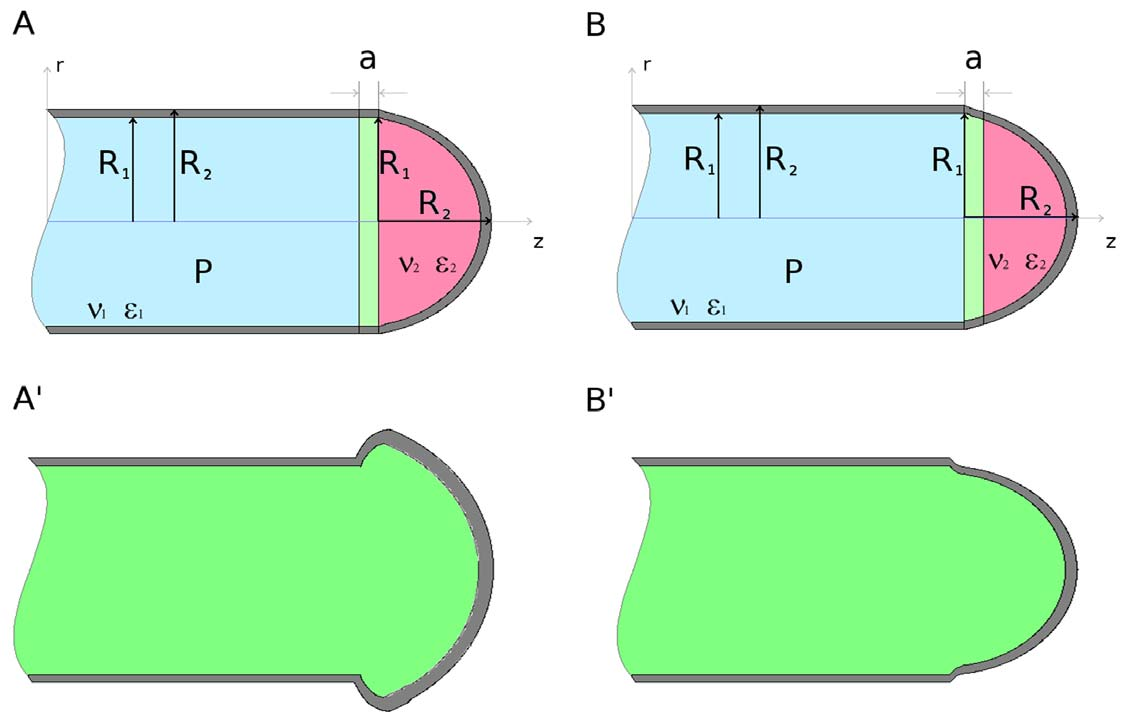
Schematic diagram of the apical region of pollen tube – the two considered axisymmetric zones: cylindrical for the distal part and semispherical for apex. (A) A narrow, symbolically denoted by ‘a’, ring of cylindrical symmetry. (B) Description as for (A) but for the spherical symmetry. (A’), (B’) – corresponding ‘breathing modes’ resulting from changing symmetries at the ‘a‘ limit (see the text).

Pollen tube growth is a crucial process in the life cycle of higher plants as it ensures the transfer of the sperm cells from the male gametophyte to the female gametophyte. It has been widely studied as a model for tip growth by plant cells (see e.g. [1] for review). The pollen tube tip is capped by an approximately hemisphere shaped dome, the apex, to which all growth activity is confined (Fig. 1A–B). It is known that pollen tubes in vitro display regular oscillations in growth velocity [2], [3] and the phenomenon is presumed to occur in vivo as well [4]. The question arises what controls pollen tube growth and what is the mechanism responsible for the growth rate oscillations. A controversy swirls around the modes of extension leading to periodicity in the growth rate. While some authors claim that hydrodynamics is the central integrator of pollen tube growth leading to growth oscillations [5], [6], [7], others couple the periodicity in growth dynamics to the changes in the wall material properties [1], [8], [9], [10].

In search for the cause of experimentally observed pollen tube growth oscillations we link exact stress/strain (analytic) relations with the wall mechanical properties of a tip growing cell. This is based on the observation that cell wall assembly by exocytosis occurs mainly at an annular region around the pole of the cell [6], [11] and that the concomitant turgor driven deformation of the cell wall causes characteristic strain exclusively in the hemisphere shaped apex of the cell [12], [13], [14]. The description of the dynamical properties of such a complex growing system should be solved self-consistently, meaning that the turgor pressure and the wall properties are conjugated variables and the resulting equations have to be solved iteratively. Dumais et al. [15] presented an anisotropic-viscoplastic model of plant cell morphogenesis by tip growth. The authors offered three sets of equations whose solutions demonstrate the importance of cell geometry, and of wall stresses/strains in the study of plant cell morphogenesis and growth. Intriguingly, the pollen tube geometry can be described by two different symmetries - a cylindrical shank region, and a hemisphere shaped apex - that are connected by a transition zone linking the two portions. In the present paper, we propose a mechanism of ‘symmetry frustration’ (MP) occurring in this transition zone between the two involved symmetries as a possible mechanism responsible for growth oscillations. At points where symmetry frustration occurs, one cannot distinguish either of the intervening symmetries. In a kind of entangled state either or both symmetry(ies) can be realized by the system. In all calculations we assume the fundamental view that wall extension is primarily a biophysical process [16], [17], although ultimately regulated by enzymatic activity, and that under conditions where the enzymatic background can be subtracted the biophysical process still proceeds normally.

It has been described by numerous groups that growth oscillations in pollen tubes are accompanied by oscillations in cellular features such as ion fluxes and signaling processes whose oscillation frequencies are identical with that of the growth rate, but whose peaks are phase delayed [3], [18], [19]. However, the roles of these cellular processes are beyond the scope of this paper and we will not discuss them here. Nevertheless, the outlined scenario leaves space for the periodic ion and mass fluxes in and out of the cell which can be considered either as upstream or downstream events to the central mechanical event.

## Model Calculations and Results

**Figure 2:**
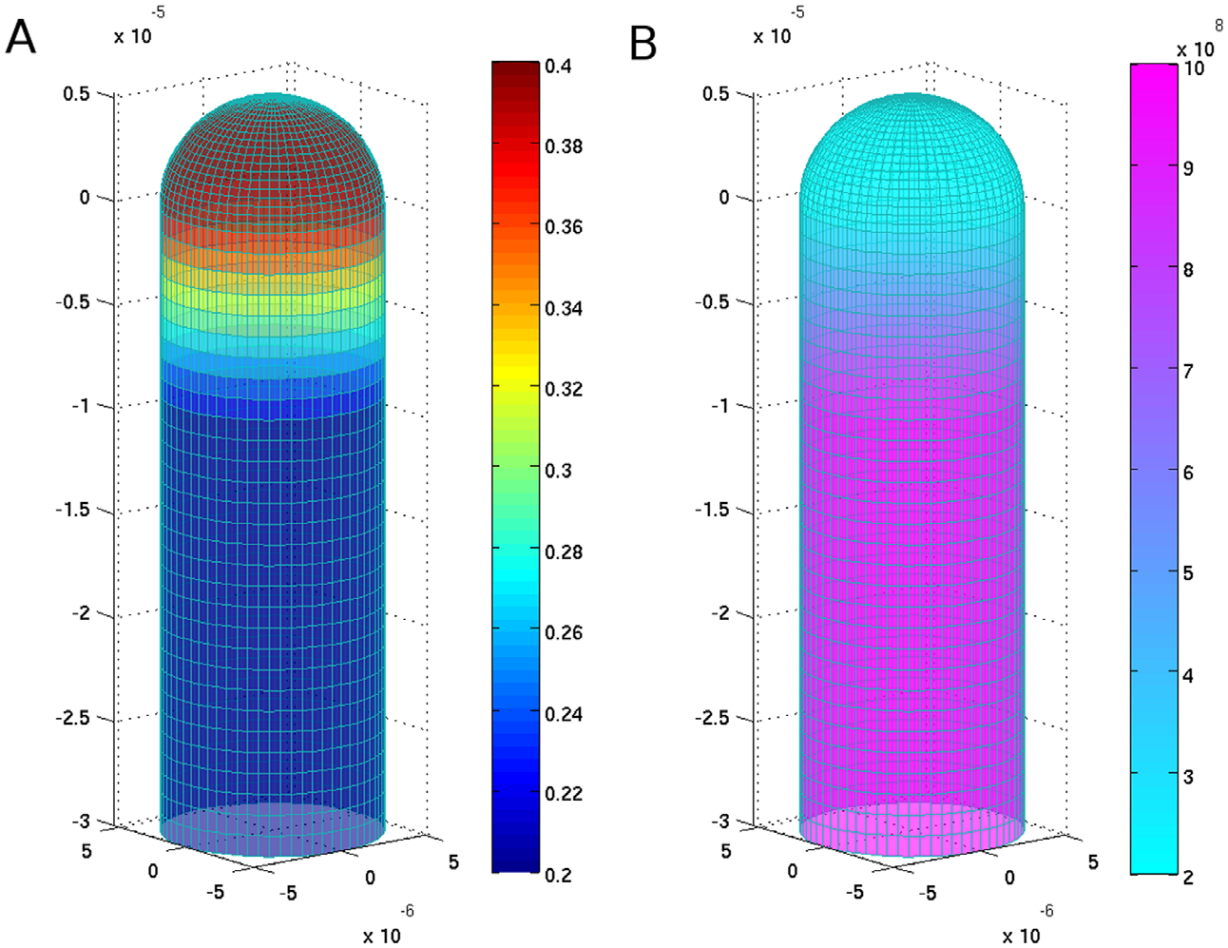
Spatial distribution of mechanical properties in the apex and apical shank of the pollen tube. (A) Poisson coefficient and (B) Young’s modulus [Pa].

The model focuses on wall mechanical properties, system pressures, analytic stress/strain relations and involved symmetries, and is proposed as follows. The apical region of the pollen tube is considered as a hollow thin-walled cylinder capped with a hemispherical tip (Fig. 1A–B). The inner radius of the cylinder equals *R_1_*, while the outer radius equals *R_2_* (the same description holds for the hemispherical dome). The mechanical properties of the wall are described by two constants: Poisson coefficient ν and Young’s modulus ε. Based on data by Geitmann and Parre [20], Fayant et al. [12], Rojas et al. [14], we set the (ν, ε) pair to differ for the shank (rigid) and the tip (elastic), respectively (Fig. 2). We assume the hydrostatic pressure *P* as the effective pressure equilibrating mechanical wall stress in the system. In future implementations, we may also include an additional osmotic pressure ΔΠ when considering different osmotic environments (hypertonic, isotonic, hypotonic). The yield threshold *Y*, usually present in cell growth description [21], [22], is omitted since it is considered as the another material constant, already present in the wall mechanical description. Finally, in the calculations we assume cylindrical geometry (and hence cylindrical symmetry) for the tubular shank, and hemispherical geometry (spherical symmetry) for the apical region of the growing pollen tube.

According to classical textbooks by Landau and Lifshitz [23], the equilibrium equation for the displacement vector 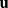 takes the form:

**Figure 3:**
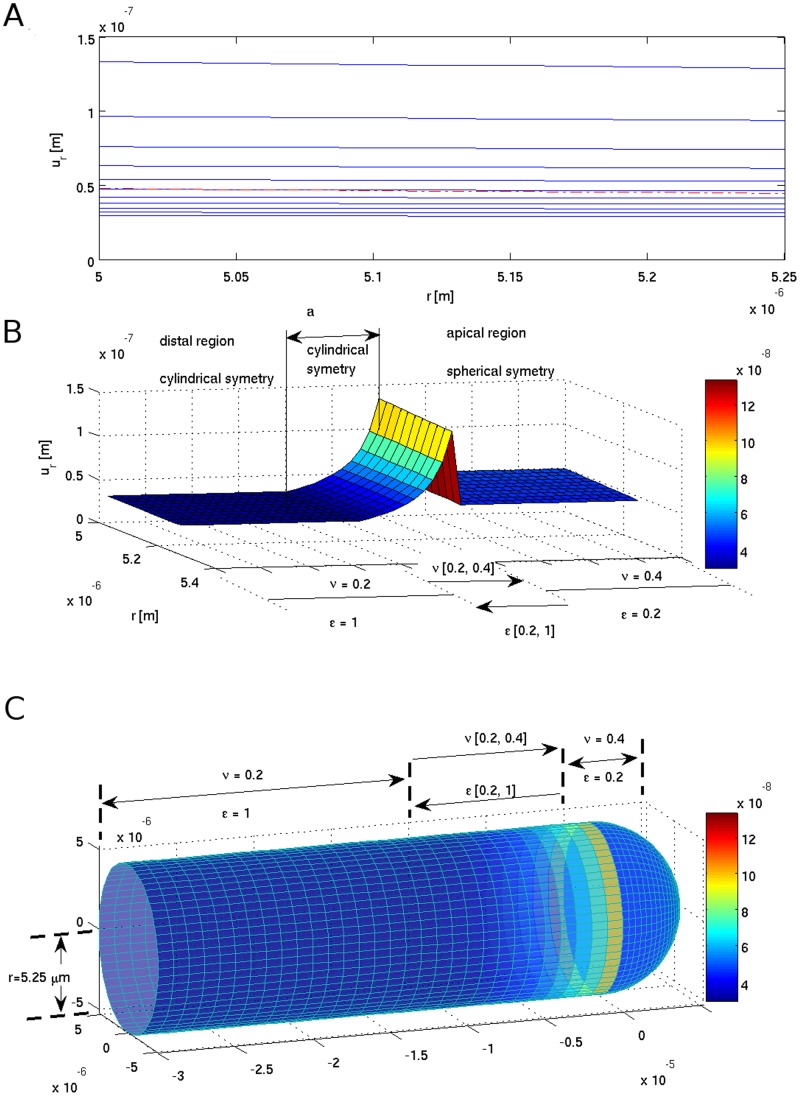
Tensile strain 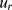 (A and B) due to the turgor pressure *P* acting on the cell wall as a function of continuously changing wall mechanical parameters (indicated in the Chart) - cylindrical symmetry at the transition zone. Radial part of the tensile strain 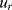 (color bar) visualized on the pollen tube surface (C). Symmetry change induces frustration at the transition ‘a’ zone (A) – a red (dash-dotted) line immersed in the blue background.



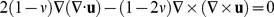
(1)where 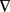 is the nabla differential operator and ν is the Poisson coefficient. We assume that the distribution of these properties is radially symmetric and that they only change in meridional direction (along the *z*-axis). Interestingly, we note that acting divergence operator (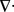) on Eq. (1) yields 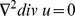, which means that the volume change due to displacement field satisfies Laplace’s equation.

Eq. (1) may be solved analytically, providing that a problem exhibits high degree of symmetry. In particular it can be solved exactly for spherical 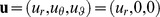 and cylindrical 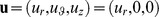 symmetries, both of which are present in the hemispherical apex and the subapical cylindrical shank of the pollen tube. This fact can be used to solve Eq. (1) and for further description of the pollen tube tip-shape and dynamical properties.

**Figure 4:**
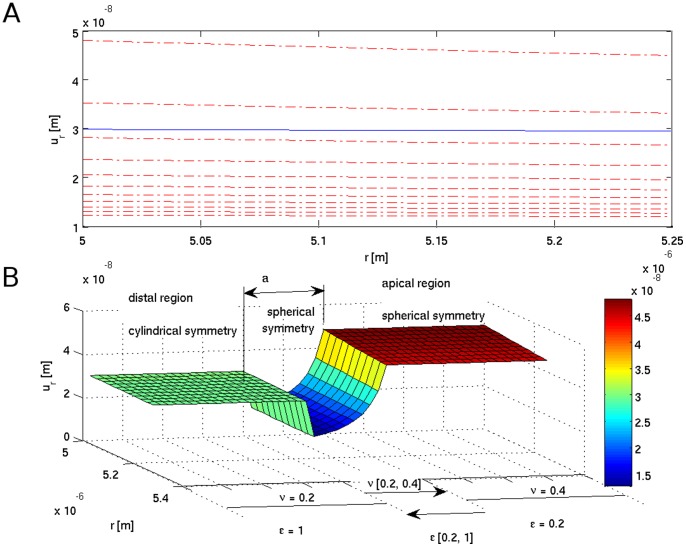
Tensile strain 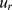 due to the turgor pressure *P* acting on the cell wall as a function of changing wall mechanical parameters (indicated) - spherical symmetry at the transition region. Symmetry change induces frustration at the transition ‘a’ zone (A) visible as blue (solid) line immersed in the red background.

By assuming cylindrical geometry for the shank, and representing field operators (grad, div and curl) in Eq. (1) in cylindrical coordinates, Eq. (1) can be presented in a much simpler form:
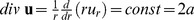
(2)


**Figure 5:**
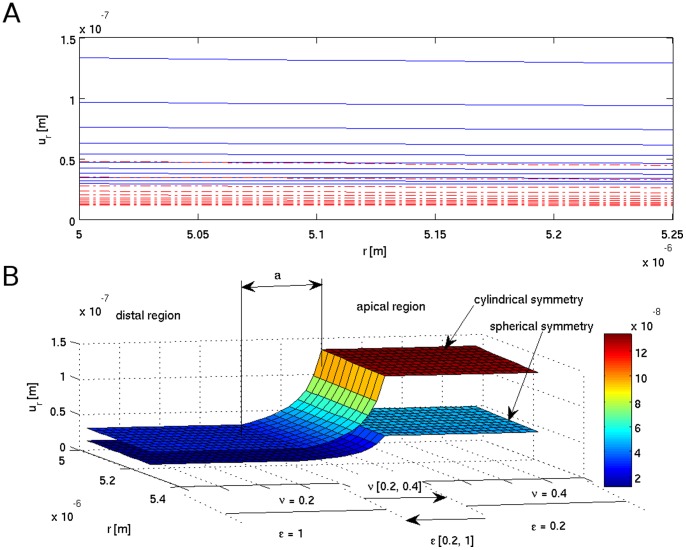
Tensile strain 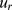 due to the turgor pressure *P* acting on the cell wall as a function of changing wall mechanical parameters (indicated) of a given symmetry (B). Different symmetry stripes show intermingling of tensile strain lines (mixed red and blue area) causing frustration (A).

Hence the solution
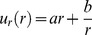
(3)represents radial deformation of a hollow cylindrical tube under pressure *P.* Parameters *a* and *b* are to be determined from the boundary conditions and 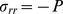 for 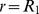 and 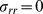 for 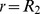. They both read:
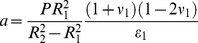
(4)

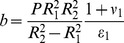
(5)


**Figure 6:**
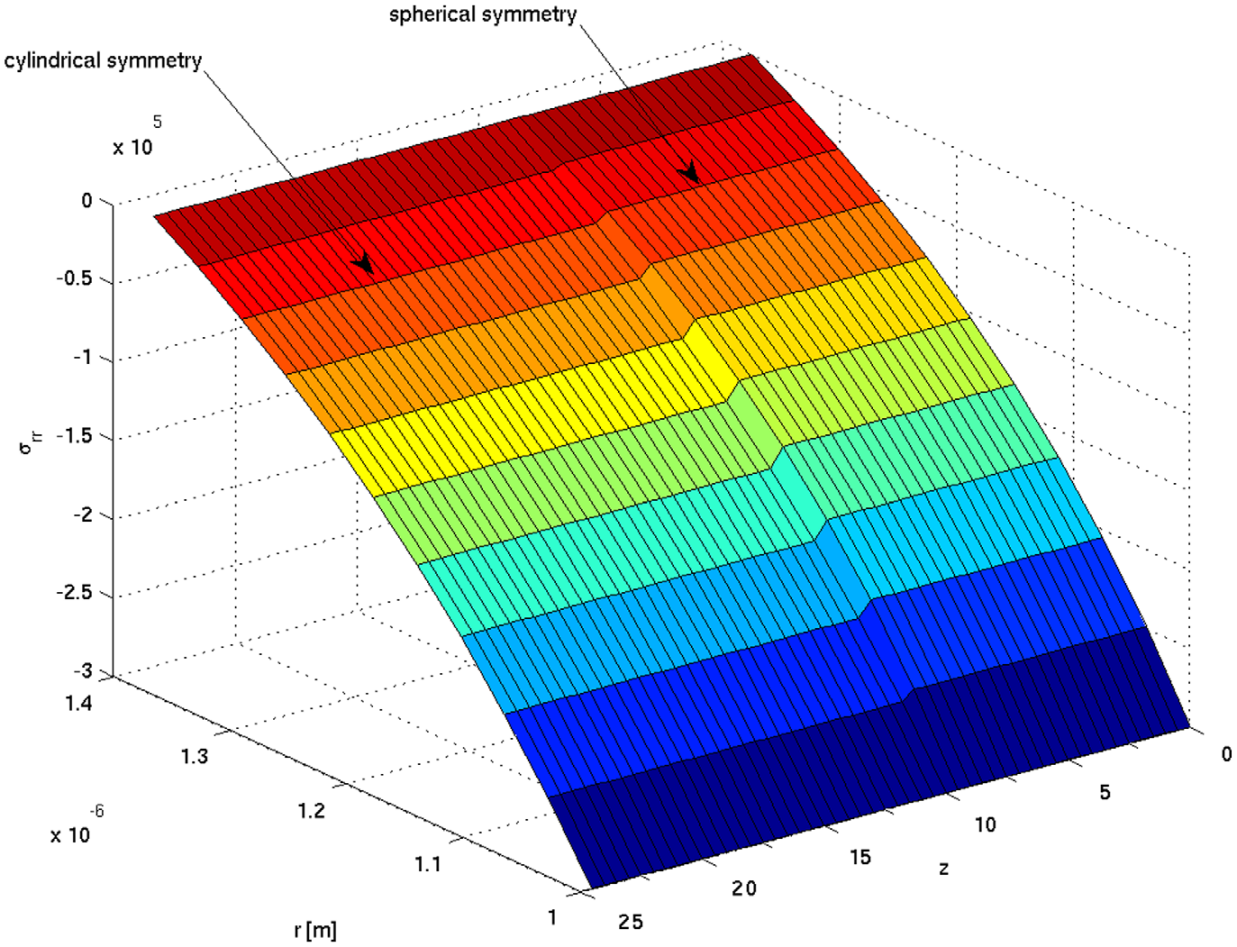
Radial stress tensor σ_rr_ [Pa] as a function of radial distance *r* from the symmetry axis (pointing in *z* direction) and meridional distance *z* from the tip. A clearly visible step in stress manifold induced solely by the symmetry brake from the spherical to the cylindrical one. Calculation parameters are the same for cylindrical and spherical symmetries. Turgor pressure *P* = 0.3 MPa. Radial distance 1–1.4 µm is taken in this case to better visualize the step in the stress tensor.

Stress distribution with respect to the cylinder cell wall thickness under pressure *P* equals:
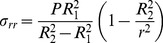
(6)


**Figure 7:**
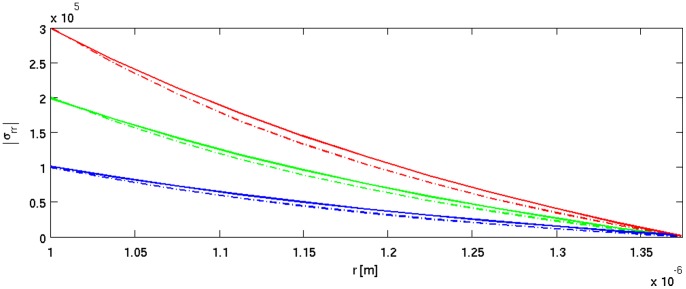
Tensile stress σ_rr_ [Pa] calculated for cylindrical (solid line) and spherical (dashed line) symmetries parameterized by turgor pressure: red –0.3 MPa, green –0.2 MPa, blue –0.1 MPa. Radial distance 1–1.4 µm is taken to better visualize the differences (solid and dash-dotted lines) in the stress tensor.

Next, by assuming spherical symmetry for the tip we introduce a spherical coordinate system with the origin in the center of the sphere. Since the deformation is solely a function of the radius *r*, hence 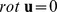 in Eq. (1) and we end up with 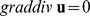. In spherical coordinates we have
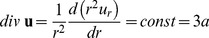
(7)with the solution for the radial displacement in the spherical symmetry given by
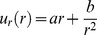
(8)where both parameters a and b can be obtained from the boundary conditions 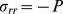 for 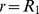 and 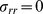 for 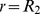, similar as for the cylindrical case. The distribution of stress in the spherical shell with inner pressure P (and outer pressure zero) reads:



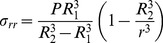
(9)The simulation results are presented in Figs. 3, 4, 5, 6, 7, and in the Supporting Information files. In Figures 3, 4, 5 the dependence of 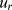 on the radius *r* is visualized. In order to illustrate the effect of symmetry change in the transition zone, we simulated the radial strain caused by the turgor pressure acting as a function of spatially changing Young's modulus and Poisson ratio in two situations. First, the transition region ‘a’ was considered to be part of the cylindrical shank (Fig. 3), second, the transition region was considered to be part of the apex (Fig. 4). Assuming cylindrical symmetry for the transition region, the simulations predict a ‘kink-like’ behavior with a local peak in radial strain at the transition region (Fig. 3). This situation corresponds to Fig. 1A’ and is consistent with temporal pollen tube swelling observed by Rojas et al. [14] and Zonia and Munnik [24]. When spherical symmetry for the transition region is assumed, the radial strain exhibits a ‘narrow well’ in the transition region (Fig. 4). This situation corresponds to Fig. 1B’ and is consistent with the narrowing of the pollen tube apex occurring during the growth cycle [14]. A ‘bifurcation-like’ diagram is shown in Fig. 5. where the radial tensile strain 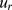 due to the turgor pressure *P* acting on the cell wall as a function of changing wall mechanical parameters for a given symmetry is presented. Stripes of both considered symmetries differ along the ‘z’-axis (meridional direction). Each stripe presents the behavior of the strain in radial direction for only one kind of symmetry. The upper stripe presents the strain 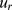 for the cylindrical symmetry – the shank behavior is extended for the whole apical region and the mechanical parameters of the wall change. Accordingly, the lower stripe presents the strain 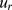 for spherical symmetry as if it were extended for the whole apex into the subapical tubular shank. As it is shown in Fig. 5 there are no common points in the strain 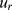 for different symmetries. Therefore, if the symmetry changes a transition between the strain stripes must appear.

The radial component of the stress tensor σ_rr_, as a function of the radial distance *r* from the symmetry axis (pointing in *z* direction) and axial distance *z* from the tip, is presented in Fig. 6. Even though we use the same mechanical parameters for both considered symmetries, a discontinuity in the stress manifold appears. The step is induced solely by the symmetry change from the spherical (apex) to the cylindrical (shank) one. In Fig. 7 the tensile stress calculated for cylindrical and spherical symmetries parameterized by turgor pressure *P* is shown.

In order to simulate the growth process, we assume that growth is proportional to the radial part of the strain tensor 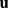 at the transition zone ‘a’. The wall is stretched in radial direction proportional to 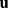 and as a result, the wall becomes thinner with respect to the adjacent regions both in distal and apical direction. Due to a reduction in tensile resistance, this thinning in turn is assumed to cause this wall section to yield in *z* direction. Simultaneously, assembly of new cell wall material by exocytosis is assumed to compensate for wall thinning and reestablishes the original wall thickness in the transition zone. Movie S1 illustrates the resulting steady growth process when cylindrical symmetry is assumed in the transition zone and pressure is applied. In this situation, the growth in ‘z’ direction is proportional to the strain for cylindrical symmetry. The same can be done for spherical symmetry in the transition zone (Movie S2). Oscillations similar to those observed by Rojas et al. [14] would then be generated by the repeated change of growth rate and cell shape resulting from an alternation between the two possible symmetries in the transition zone. Because the strain 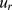 is smaller for the spherical symmetry, growth is slower in this situation compared to the cylindrical symmetry. The 'lower order' cylindrical symmetry is clearly more efficient to ensure elongation growth compared to the spherical symmetry.

## Discussion

Polar growth in pollen tubes is associated with spatially confined dynamic changes in cell mechanical properties [20], [25]. This confirms the important role of the mechanical properties of the cell wall for local cellular growth processes in plants [26]. It was shown experimentally that the initial formation of a cylindrical protuberance is preceded by a local reduction in cellular stiffness [25]. Such softening was also registered before a rapid growth phase in cells with oscillating growth pattern [25]. The local soft spot at the apical cell wall is maintained by the continuous insertion of new cell wall material [27]. This insertion occurs over the entire apex, but is thought to be highester in a ring-shaped region around the pole of the cell [11], [27], [28], [29], [30]. The delivery is mediated by secretory vesicles transported on an actin array, the subapical ‘actin fringe’, the proximal end of which reaches into the apical cytoplasm [31], [32]. Starting with its insertion into the apoplastic space by exocytosis, the pectic cell wall material matures by de-methyl esterification which results in an increased stiffening mediated by calcium gelation of the pectin monomers [12], [20]. The unstiffened, viscoplastic cell wall material at the apex is mechanically deformed driven by the hydrostatic turgor pressure resulting in the continuous elongation of the pollen tube. Once the cell wall material is stiffened it ceases to be deformed by the turgor and forms the cylindrical shank of the tube.

In other models, the ageing process of the cell wall is considered as a kinetic process [33], [34]. Here we simply impose a spatial gradient of mechanical properties to investigate the role of geometry in determining the strain pattern resulting from the internal turgor pressure. The only assumptions on which our model is based are the quantitative details of the gradients of cytomechanical properties (Young's modulus and Poisson coefficient). We furthermore assume that any stretch induced thinning is compensated by insertion of new cell wall material. Both assumptions are justified by experimental data obtained by others. The remaining model ingredients were delivered by exact solutions for pollen tube specific geometry derived from the equation of equilibrium, Eq. (1).

Our simulations are consistent with the widely accepted notion that cell shape in the pollen tubes is regulated by the mechanical properties of the cell wall, whereas turgor only serves as the driving force for expansion [1], [10]. They illustrate that temporal changes in turgor are not necessary to explain oscillatory shape changes during pollen tube growth as postulated by others [35]. We clearly demonstrate that the transition region between apical hemisphere and cylindrical shank of the tube is characterized by symmetry frustration which causes the local radial strain to display a significant peak in this transition zone. As a result, we propose that this peak in radial strain is translated into longitudinal strain caused by the local thinning and hence loss in tensile resistance of the cell wall. We assume that continuous assembly of new cell wall material in the apex compensates for this stretch-induced thinning. The fact that depending on whether spherical or cylindrical symmetry is applicable for the transition zone, the strain rates are different opens intriguing avenues to investigate the implications of this phenomenon for oscillatory growth. The turgor pressure induces the radial strain 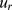 (wall thinning). Assuming that this is followed by a change of symmetry, wall extension in z-direction appears as a result of stress relaxation. This phenomenon is independent of the direction of the symmetry change. If the symmetry of transition region ‘a’ changes from cylindrical to spherical (see also Fig. 1A and A'), insertion of new cell wall material would have to proceed on the shank side of the transition zone to compensate for strain induced thinning, while if the symmetry changes from spherical to cylindrical (Fig. 1B and B'), cell wall insertion would proceed on the dome side of the transition zone. These changes of symmetry hence and forth produce fluctuations which from the macroscopic point of view can be observed as growth cycles.

Since the wall stress σ_rr_ is directly proportional to the constant turgor pressure *P* (Eqs (6) and (9)), osmotically induced turgor pressure changes would be predicted to alter the stress in the wall. It could be argued that this is analogous to a violin string: the higher the tension, the higher the pitch. If this is the case, higher wall stress will cause higher frequencies of oscillations. This is in agreement with the experimentally observed frequency increase in the transition from low pressure hypertonic, through intermediate pressure isotonic, to high pressure hypotonic conditions [5].

An interesting prediction of the present model is the inherent instability of the transition zone linking the hemisphere shaped apex and the tubular shank. To validate this experimentally, quantitative measurement of the local mechanical properties of the cell wall are required at a spatial resolution above that published hitherto [20]. These should reveal a local change in mechanical properties that coincides with the transition region ‘a’. Further evidence for the predicted location of instability is provided by the observation that bursting pollen tubes almost always rupture at the transition zone - independently of the trigger that caused the event (Movie S3).

An oscillatory changing stress situation in the pollen tube apex as predicted by our model could also be the mechanical trigger that is responsible for opening and closing ion channels in the apical region. The presence of stretch-activated calcium channels has been proposed to be responsible for temporal increases in calcium influx and, consequently, in cytosolic calcium concentration [8]. Indeed, in accordance with Fig. 5, a tensile strain in the apex of a pollen tube changes due to symmetry change and the magnitude of the change is proportional to the distance between the two layers shown in Fig. 5.

The changes in the magnitude of strain occurring at and near the transition zone also opens intriguing paths for the question of how polarity is maintained. Numerous molecules are associated exclusively with the apical plasma membrane or cell wall of the pollen tube [36]. For example, the pectin methyl esterase inhibitor is associated only with the apex and has been postulated to be endocytosed in a region that corresponds exactly to the transition region [37]. Since membrane tension affects both exocytosis and endocytosis [38], local and steep changes in the strain profile may provide a possible explanation why endocytosis takes place exactly here.

### Conclusions

We based our structural model on the parameterized description of a tip-growing cell that allows the manipulation of cell size, cell shape, cell wall thickness, cell geometry, gradient of the local mechanical properties (spatial distribution of the Young’s modulus and Poisson coefficient) and turgor pressure. Although based on simple geometrical considerations, the model is able to explain a number of experimental findings that have puzzled biologists and even caused controversies. It explains why pollen tubes typically rupture between the apex and the shank rather than at the very tip of the cell. It provides an explanation for variations in cell diameter at constant turgor, and it suggests that experimentally observed growth oscillations may have a geometrical component that should be taken into account when analyzing the cell biological underpinnings of the process. Experimental research is warranted to further investigate the possible implications of symmetry frustration in the elongation of tip growing cells.

## Supporting Information

Movie S1Simulation of expansive growth in the apical region of the pollen tube assuming cylindrical symmetry at transition zone ‘a’. In the simulation, we assume that growth is proportional to the radial part of the strain tensor 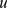 (color bar). The wall is stretched in radial direction proportional to 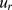 causing a local thinning which in turn results in expansion in z direction. Prior to the subsequent application of pressure, wall thinning is assumed to be compensated for by delivery of new cell wall material. 20 growth cycles are presented.(MPG)

Movie S2Simulation of expansive growth in the apical region of the pollen tube assuming spherical symmetry at transition zone ‘a’. In the simulation, we assume that growth is proportional to the radial part of the strain tensor 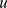 (color bar). The wall is stretched in radial direction proportional to 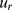 causing a local thinning which in turn results in expansion in z direction. Prior to the subsequent application of pressure, wall thinning is assumed to be compensated for by delivery of new cell wall material.20 growth cycles are presented.(MPG)

Movie S3 Elongating pollen tube explodes after addition of 0.1 mM Gd^3+^, an inhibitor of calcium channels. The site of rupture is at the transition region, at the base of the hemisphere shaped apex.
(AVI)
